# Metastatic lymph node ratio demonstrates better prognostic stratification than pN staging in patients with esophageal squamous cell carcinoma after esophagectomy

**DOI:** 10.1038/srep38804

**Published:** 2016-12-12

**Authors:** Hongdian Zhang, Huagang Liang, Yongyin Gao, Xiaobin Shang, Lei Gong, Zhao Ma, Ke Sun, Peng Tang, Zhentao Yu

**Affiliations:** 1 Department of Esophageal Cancer, Tianjin Medical University Cancer Institute and Hospital, Key Laboratory of Cancer Prevention and Therapy of Tianjin City, Tianjin 300060, China; 2 Department of Thoracic Surgery, The First Hospital of Qinhuangdao, Hebei 066000, China; 3 Department of Cardiopulmonary Function, Tianjin Medical University Cancer Institute and Hospital, Key Laboratory of Cancer Prevention and Therapy of Tianjin City, Tianjin 300060, China

## Abstract

This study aimed to evaluate the prognostic significance of lymph node ratio (LNR) by establishing a hypothetical tumor-ratio-metastasis (TRM) staging system in patients with esophageal squamous cell carcinoma (ESCC). The records of 387 ESCC patients receiving curative esophagectomy were retrospectively investigated. The optimal cut-point for LNR was assessed via the best cut-off approach. Potential prognostic parameters were identified through univariate and multivariate analyses. A novel LNR-based TRM stage was proposed. The prognostic discriminatory ability and prediction accuracy of each system were determined using hazard ratio (HR), Akaike information criterion (AIC), concordance index (C-index), and area under the receiver operating characteristic curve (AUC). The optimal cut-points of LNR were set at 0, 0~0.2, 0.2~0.4, and 0.4~1.0. Multivariate Cox analysis indicated that the LNR category was an independent risk factor of overall survival (*P* < 0.001). The calibration curves for the probability of 3- and 5-year survival showed good consistency between nomogram prediction and actual observation. The LNR category and TRM stage yielded a larger HR, a smaller AIC, a larger C-index, and a larger AUC than the N category and TNM stage did. In summary, the proposed LNR category was superior to the conventional N category in predicting the prognosis of ESCC patients.

Esophageal cancer (EC) is the third most frequently malignant tumor and the fourth leading cause of cancer-related deaths in China^1^. Radical esophagectomy with lymphadenectomy has been applied as the major treatment modality for resectable EC. Despite the improvements in multimodal treatment strategies in the past decades, the prognosis of EC remains poor.

The status of regional lymph node (LN) metastasis has been widely considered as an important prognostic factor to plan subsequent postoperative treatments for patients with EC. In 2009, the 7th edition International Union Against Cancer (UICC)/American Joint Committee on Cancer (AJCC) TNM staging system proposed an updated pathologic nodal category on the basis of the number of metastatic LNs^2^. Although the proposed category is a simple, convenient, and reliable method for precise staging, the identified number of metastatic LNs depends on the number of dissected LNs. A low number of examined LNs may lead to understaging and subsequent underestimation of disease severity, and this phenomenon is referred to as stage migration^3^.

An absolute standard or guideline regarding the number of LNs to be dissected for accurate staging has yet to be developed. According to the 7th AJCC TNM staging system, at least 12 LNs should be retrieved to develop accurate N staging^4^. Yang *et al*.^5^ and Greenstein *et al*.^6^ suggested 18 nodes as the minimum number of dissected LNs to obtain an accurate staging. Peyre *et al*.^7^ recommended that at least 23 regional LNs should be examined in 1,053 patients from nine EC centers.

Lymph node ratio (LNR), which is calculated as the ratio of the number of metastatic LNs to the total number of removed LNs, has been proposed to address problems related to the variability of nodal examination. It has been confirmed as a highly reliable indicator used to evaluate the prognosis of various malignant solid tumors, such as lung, breast, gastric, colon, gallbladder, and ovarian cancer^8^,^9^,^10^,^11^,^12^. Although the advantage of LNR over the AJCC N category in predicting the prognosis of patients with EC has been extensively explored, this parameter has yet to be integrated into the current staging system^13^.

In the light of these considerations, our study was conducted to determine the staging standards of LNR and to compare this ratio with the pathological N category for the prognostic evaluation of esophageal squamous cell carcinoma (ESCC). We also established an optimal tumor-ratio-metastasis (TRM) staging system and investigated whether the hypothetical staging system can be more accurately applied to predict the prognosis of ESCC patients than traditional TNM staging system.

## Results

### Patient demographics

The clinicopathological parameters of the 387 ESCC patients included in our study are summarized in Table 1. There were 320 (82.7%) males and 67 (17.3%) females with a median age of 68 years (range 39 years to 99 years). Of these patients, 271 (70.0%) had smoking history. Most of the primary tumor sites were middle thorax (n = 314 [81.1%]) and lower thorax (n = 52 [13.4%]). According to histological grade, 33 (8.5%) patients were well differentiated, 294 (76.0%) were moderately differentiated, and 60 (15.5%) were poorly differentiated/undifferentiated. The average largest tumor diameter was 4.2 cm (median: 3.8 cm, range, 0.5 cm to 12.0 cm).

All patients underwent Ivor-Lewis surgical resection. Among the cohort, the average number of total retrieved LNs per patient was 15.2 (range, 4 to 58). According to the 7th edition AJCC TNM staging system, 223 (57.6%), 99 (25.6%), 35 (9.0%), and 30 (7.8%) patients were classified as N0, N1, N2, and N3, respectively. As regard to the TNM staging system, 45 (11.6%) patients were in stage I, 158 (40.8%) patients were in stage II, 102 (26.4%) patients were in stage IIIA, 19 (4.9%) patients were in stage IIIB, and 63 (16.3%) patients were in stage IIIC.

### Correlation of the number of retrieved nodes, metastatic nodes, and LNR

**Figure 1:**
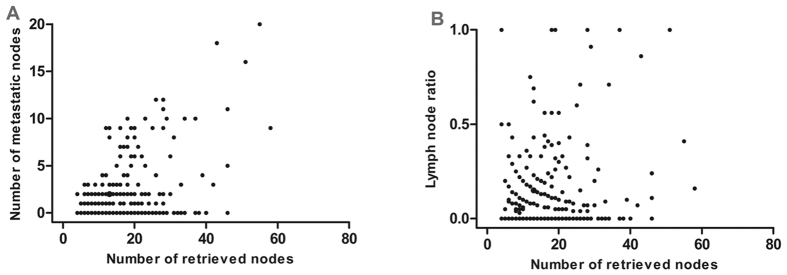
Spearman’s correlation analyses among the number of retrieved lymph nodes, metastatic lymph nodes, and lymph node ratio. (**A**) Significant correlation between the number of retrieved lymph nodes and the number of metastatic lymph nodes (*r* = 0.204, *P* < 0.001). (**B**) No significant correlation between the number of retrieved lymph nodes and lymph node ratio (*r* = 0.091, *P* = 0.073).

Spearman’s correlation analysis showed that the total number of retrieved LNs was significantly related to the number of metastatic LNs (*r* = 0.204, *P* < 0.001, Fig. 1A), whereas the number of retrieved LNs was not correlated with LNR (*r* = 0.091, *P* = 0.073, Fig. 1B). These results demonstrated that LNR was not influenced by surgery, but pN was influenced by surgery.

### Cut-point survival analysis for the determination of the optimal cut-point of LNR

As described in the “Methods”, the patients without LN metastasis were classified as LNR0 (0%). Furthermore, the other patients were stratified into five groups by every 0.20 interval of LNR. According to the best cut-off approach by the log-rank test, the survival rates for the categories 0.41~0.6, 0.61~0.8, and 0.81~1 were similar. So we divided the LNR into four subgroups as follows: LNR 0, 0% (n = 223); LNR 1, 1~20% (n = 95); LNR 2, 21~40% (n = 38); LNR 3, >40% (n = 31). Accordingly, a novel TRM staging was established: 47 (12.1%) patients were in stage I, 150 (38.8%) patients were in stage II, 105 (27.1%) patients were in stage IIIA, 20 (5.2%) patients were in stage IIIB, and 65 patients (16.8%) were in stage IIIC.

### Univariate survival analysis

**Figure 2:**
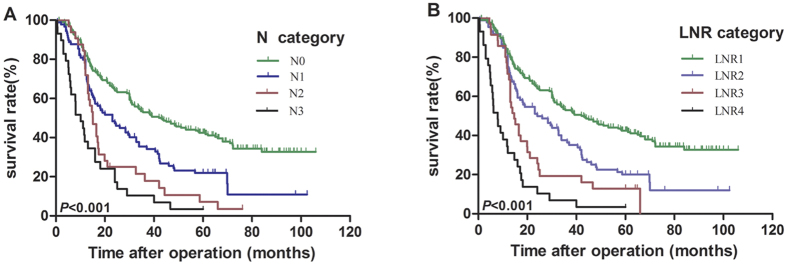
Effect of the (**A**) N category and (**B**) LNR category on the prognosis of ESCC patients after radical esophagectomy (*P* < 0.001; Kaplan-Meier and log-rank test).

The 1, 3, 5-year overall survival (OS) rates of the entire cohort were 78.6%, 42.4% and 31.2%, respectively, and the median OS time was 29.5 months. For patients classified as N0, N1, N2, and N3 according to the AJCC N category, the 5-year OS rates were 42.5%, 21.9%, 7.1%, and 3.4%, respectively, and the median survival times of these four groups were 43.0, 23.0, 15.0, and 10.0 months, respectively. (N0 vs. N1, *P* < 0.001; N1 vs. N2, *P* = 0.046; N2 vs. N3, *P* = 0.065, by a log-rank test). For patients classified as LNR0, LNR1, LNR2, and LNR3, the observed 5-year OS rates were 42.5%, 20.1%, 12.9%, and 3.6%, respectively, and the median survival times of these four groups were 43.0, 24.0, 14.6, and 7.9 months, respectively.(LNR0 vs. LNR1, *P* < 0.001; LNR1 vs. LNR2, *P* = 0.035; LNR2 vs. LNR3, *P* = 0.006, by a log-rank test). The survival curves according to the AJCC N category and the LNR category are shown in Fig. 2.

The clinicopathological factors analyzed in the univariate survival analysis are also shown in Table 1. The factors significantly influencing the 5-year OS were patient age (*P* = 0.038), smoking history (*P* = 0.026), tumor size (*P* < 0.001), histological grade (*P* = 0.036), T category (*P* < 0.001), N category (*P* < 0.001) and LNR category (*P* < 0.001) after esophagectomy. By contrast, gender and tumor location did not affect OS.

### Multivariate survival analysis

Multivariate survival analysis was performed with Cox’s proportional hazard regression model to identify the independent factors correlated with prognosis. When either N category or LNR category was included in the analysis models, it was found to be one of the most significant independent prognostic factors for OS, in addition to tumor size, histological grade, and T category (*P* < 0.05 for these parameters). However, the N category (*P* = 0.309) no longer significantly predicted survival when the N category and the LNR category were simultaneously considered covariates. By comparison, the LNR category (*P* < 0.001) remained a significant indicator of prognosis (Table 2).

**Figure 3:**
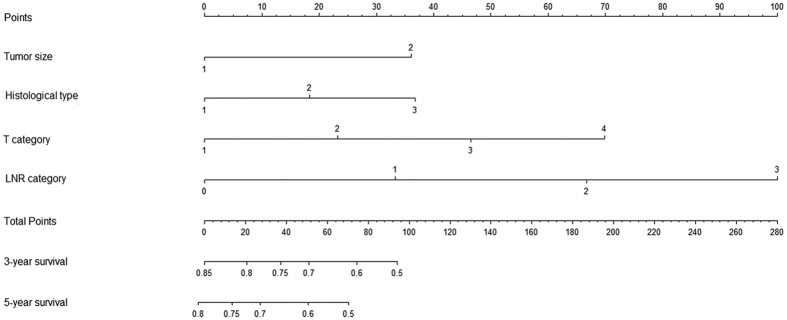
Nomogram predicting 3- and 5-year overall survival for the patients with ESCC after radical esophagectomy. (In using the nomogram, the value attributed to an individual patient is located on each variable axis, and a line is drawn upward to determine the risk score for each variant value. The sum of these scores is located on the total point axis, and a line is drawn downward to the survival axes to determine the probability of 3- or 5-year survival).

**Figure 4:**
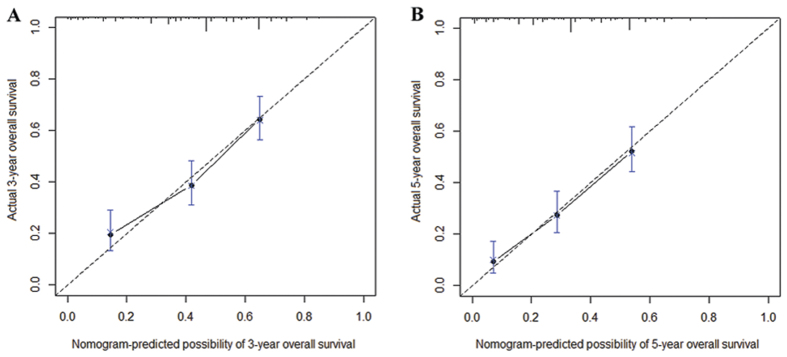
Calibration curves for predicting patient overall survival at 3- year (**A**) and 5- year (**B**) in the patients with ESCC after radical esophagectomy. The X-axis represents the nomogram-predicted survival, and the actual survival is plotted on the Y-axis. The dotted line represents the ideal correlation between predicted and actual survival.

Furthermore, nomograms were used to predict the 3- and 5-year OS of patients. LNR category was selected as an independent prognostic factor in nomograms (Fig. 3), which were similar to those in the aforementioned multivariate analyses conducted by Cox regression. Moreover, the calibration plot for the probability of survival at 3- or 5- years after surgery revealed a good correlation between the predicted survival probability and the actual survival rate (Fig. 4).

### Correlation analysis between N and LNR categories

To evaluate the prognostic performance of the AJCC N and LNR category, the 5-year OS rates of patients were compared with different N categories when stratifying by LNR, and with different LNR categories when stratifying by N category. For patients in each of the N category, significant differences in survival could be observed among patients in different LNR categories. However, for patients in each of the LNR category, the prognosis was highly homologous when the patients were classified with different N categories (Supplementary Table S1). These results indicated that the LNR category could be more precisely used to identify the subgroups of ESCC patients with similar prognosis than the N category.

### Survival analysis based on the AJCC TNM and TRM stages

**Figure 5:**
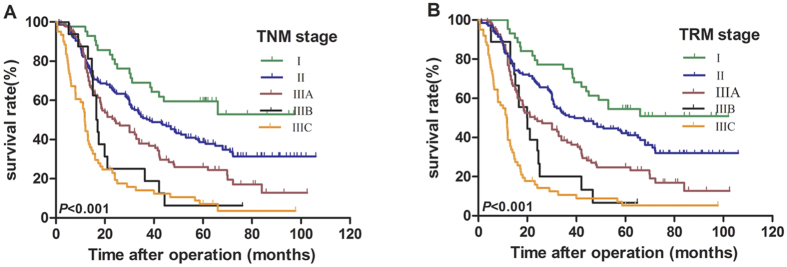
Effect of the (**A**) tumor-node-metastasis (TNM) stage and (**B**) tumor-ratio-metastasis (TRM) stage on the overall survival of ESCC patients after radical esophagectomy. (*P* < 0.001; Kaplan-Meier and log-rank test).

The 5-year OS rates of patients from stages I to IIIC in the AJCC TNM stage were 59.5%, 38.7%, 26.0%, 6.0%, and 7.0%, respectively (I vs. II, *P* = 0.019; II vs. IIIA, *P* = 0.012; IIIA vs. IIIB, *P* = 0.135; IIIB vs. IIIC, *P* = 0.178, Table 3, Fig. 5A). The 5-years OS rates of patients from stages I to IIIC in the modified TRM stage were 54.5%, 42.2%, 24.8%, 6.7% and 5.3%, respectively (I vs. II, *P* = 0.043; II vs. IIIA, *P* = 0.003; IIIA vs. IIIB, *P* = 0.125; IIIB vs. IIIC, *P* = 0.032, Table 3, Fig. 5B).

### Comparison of prognostic discriminatory ability of different staging systems for overall survival

The prognostic discriminatory ability of the aforementioned staging systems was evaluated on the basis of the hazard ratio (HR) and Akaike information criterion (AIC). We found that the HR of the LNR category was larger than that of the N category. The AIC of the LNR category was smaller than that of the N category. Furthermore, the TRM stage yielded a larger HR and a smaller AIC than the AJCC TNM stage did. Therefore, we considered that the LNR category and the TRM stage exhibited a more efficient discriminatory ability in the prediction of the prognosis of ESCC patients than the N category and TNM stage did (Table 4).

### Comparison of prediction accuracy for overall survival among different staging systems

The prediction accuracy for 5-year OS of the aforementioned staging systems was further evaluated through the Harrell’s concordance index (C-index) analysis and the time-dependent receiver operating characteristic (ROC) curves test. As shown in Table 4, the LNR category (0.721) and TRM stage (0.738) provided a larger corresponding C-index than the N category (0.713) and TNM stage (0.723) did. Moreover, the time-dependent ROC curves showed that the area under the ROC curve (AUC) (95% CI) of each staging system for OS prediction was as follows: 0.717 (0.667–0.768) for N category, 0.726 (0.676–0.776) for LNR category, 0.728 (0.665–0.781) for TNM stage and 0.744 (0.694–0.794) for TRM stage (Supplementary Figure S1). Although no significant difference was found in the prediction accuracy between N and LNR categories, and between TNM and TRM stages (*P* > 0.05), these results suggested that the TRM stage exhibited a more efficient performance with a higher prediction accuracy than the TNM stage did, and the LNR was also more accurate than the N category.

## Discussion

Despite the advancements in early detection, surgical management, and multimodality treatment, the prognosis of EC remains unsatisfactory. As such, an effective and accurate staging system for EC is of great importance to predict prognosis and implement informed decisions regarding multidisciplinary treatment^14^. Therefore, the staging system of LN metastasis must be feasible, reproducible, and accurate for prognostic stratification without stage migration.

As indicated in previous studies, the number of metastatic LNs has been confirmed as a powerful predictor of survival among patients with ESCC. However, the prognostic power is greatly affected by the total number of retrieved LNs during surgery. If the number of retrieved LNs is inadequate, down migration of the pN stage may occur; consequently, the prognosis of patients may be overestimated^15^. Standards have yet to be established regarding the exact number of LNs that should be retrieved to minimize stage migration. It states that an adequate lymphadenectomy requires the retrieval of 6 to 30 nodes for accurate staging^5^,^6^,^7^,^16^.

The concept of LNR has been proposed to solve related problems. This ratio-based nodal staging has been well investigated in gastric cancer, lung cancer, breast cancer, and other cancer types because of its simplicity and reproducibility. Although the potential prognostic value of LNR has also been evaluated in several studies on EC, no unified and well-recognized optimal cut-point for LNR has been determined in EC^13^,^17^,^18^,^19^,^20^,^21^,^22^. Divergences may result from differences in sample sizes, inclusion criteria, pathological types, evaluation standard, and statistical methods.

Greenstein *et al*.^20^ used the Surveillance, Epidemiology, and End Results database to evaluate the relationship between LNR and survival among 838 EC patients with LN metastasis. They classified the patients into three groups according to the LNR (≤0.2, 0.21~0.5, and >0.5), and found that LNR can stratify survival better than the AJCC/UICC N stage. Tan *et al*.^13^ evaluated the prognostic value of LNR in 700 ESCC patients after tri-incisional esophagectomy by performing X-tile analysis and obtained the optimal cut-off values of 0%, 1~25%, and >25%; this finding indicated that LNR was an indicator of the prognostic stratification of patients with ESCC regardless of the number of retrieved LNs. Wei *et al*.^23^ analyzed 496 cases of ESCC patients and classified the optimal cut-point of LNR as 0, 1~15%, 15~30%, and >30%. The LNR category yielded a greater prognostic value than the N category did, especially for patients with <12 LNs removed. Further analysis revealed that the application of LNR led to the identification of subgroups of patients prognosis more homogeneous than the TNM system, which was similar to our findings.

Consistent with previously reported data, our findings revealed that the number of metastatic nodes increased proportionally to the total number of dissected LNs, but the LNR was not correlated with the total number of retrieved LNs. We established the cut-point for LNR on the basis of the statistical significance observed with increasing values of 0.2 as 0~0.20, 0.21~0.40, and >0.40 by performing log-rank test. We found that the LNR category showed a clear advantage over the AJCC N category because the former was less influenced by the extent of lymphadenectomy than the latter.

In the present study, the LNR category was superior to the 7th AJCC N category because of the following reasons. (i) In univariate analysis, the log-rank χ^2^ associated with LNR was larger than that of the AJCC N category, indicating a higher statistical significance (Table 1). (ii) In multivariate analysis, either pN or LNR was an independent prognostic factor for OS individually, and the HR for the LNR category was larger than that for the N category. However, the N category lost its significance when the two covariates compared together (Table 2). Similarly, the LNR category but not the N category was proven to be the independent prognostic factor in the nomogram (Fig. 3). (iii) The calibration curve showed an optimal calibration between the nomogram prediction and the observation of the probability of 3- or 5-year survival (Fig. 4). (iv) The LNR category could be used to classify patients with different N categories into distinct prognostic groups (Supplementary Table S1). (v) Compared with the N category and TNM stage, the LNR category and TRM stage yielded a larger HR and a smaller AIC value, representing the potential superiority of the prognostic discriminatory ability of the TRM stage (Table 4). (vi) Harrell’s C-index analysis and the time-dependent ROC curves showed larger C-index and AUC for 5-year OS prediction in the LNR category and TRM stage compared with the N category and TNM stage, which suggested that the TRM stage exhibited higher accuracy in predicting survival than the TNM stage did (Table 4 and Supplementary Figure S1).

Several limitations should be considered in this study. Firstly, this was a retrospective study involving a relatively small sample population from a single institution. Secondly, all patients included in our study underwent Ivor-Lewis esophagectomy only, and the mean number of removed LNs was 15.2. This relatively scarce LN collection result can be considered as a drawback. Thirdly, the effect of various treatment-related outcomes could not be evaluated fully in this study. None of the patients received preoperative radiotherapy or chemotherapy, and further studies should be conducted to evaluate whether the TRM stage is applicable to patients receiving neoadjuvant treatments. Despite these limitations, our results indicated that the ratio based staging system can be considered as a more reliable system to predict ESCC patient prognosis than the traditional TNM stage.

In conclusion, our investigation demonstrated that the LNR category may be a potentially convenient and reproducible prognostic variable to reduce stage migration. The novel TRM staging system based on LNR should be considered as an alternative to the current TNM staging system. Nevertheless, further investigations with a larger sample size and randomized prospective design from multicenter studies should be performed to overcome the limitations of this study and to confirm the prognostic value of LNR and the modified TRM staging system.

## Patients and Methods

### Patient eligibility

We retrospectively reviewed a cohort of 387 patients who manifested histologically confirmed primary ESCC and underwent curative esophagectomy with LN dissection in our institution from January 2005 to December 2009. Curative resection was defined as the complete tumor removal with no macroscopic residual tumor, no invasion of carcinoma cells at any resection margins, and no evidence of distant metastasis. The inclusion criteria of the study were as follows: thoracic esophageal squamous cell carcinoma, radical resection, no combined malignancy, no distant metastasis, and no preoperative chemotherapy and/or radiotherapy. On the basis of these criteria, we included 387 patients with ESCC in the analysis. This study was approved by the Research Ethics Committee of Tianjin Medical University Cancer Institute. The methods were carried out in accordance with the relevant guidelines and regulations. All of the patients signed an informed consent.

All of the patients were clinically staged through physical examinations, laboratory tests, barium esophagography, cervical and abdominal ultrasonography, computed tomography scans from the neck to the upper abdomen, upper gastrointestinal endoscopy, and tumor biopsy. Endoscopic ultrasound and positron emission tomography were later added to the staging workup. Patients were considered for surgical resection if the preoperative evaluation revealed no evidence of distant metastases and if the airway or major vascular structures were not directly invaded.

All of the patients underwent a standardized transthoracic Ivor-Lewis esophagectomy with a systematic two-field (mediastinal and abdominal) LN dissection as described previously. Gastric conduit was used as a reconstruction substitute in all of the patients. Cervical lymphadenectomy was not systematically performed. All of the removed tumor specimens and retrieved LNs were sent fresh for pathological examination by two pathologists, at least one being a specialist upper gastrointestinal pathologist. Histological grade was defined as well differentiated, moderately differentiated, or poorly differentiated, according to the World Health Organization classification of esophageal tumors^24^. All of the patients included in this study were staged on the basis of the 7th edition AJCC TNM staging criteria for ESCC^4^.

### Lymph node classifications

Lymph node metastasis was classified according to the 7th edition of the AJCC N category based on the number of metastatic lymph nodes: N0, no metastasis; N1, 1~2 metastatic LNs; N2, 3~6 metastatic LNs; and N3, ≥7 metastatic LNs. The LNR is calculated as the ratio of the number of metastatic LNs to the total number of retrieved LNs. In the present study, our analysis was conducted as follows to determine the appropriate cut-point of the LNR that determines the greatest actuarial survival difference among the resulting subgroups in the entire cohort. Patients without LN metastasis (LNR = 0) were initially assigned to one group because their prognoses significantly differed from patients with metastatic LNs. The intervals of LNR categories were subsequently determined by comparing the OS rates on the basis of LNR with an initial interval of 0.2 and then combining the neighborhood survival curves by using the log-rank test.

We developed a modified TRM staging system, which was regarded as a combination of the T category, LNR category, and M stage, to elucidate the contribution of LNR to the accuracy of the prognosis of ESCC patients. Generally speaking, the TRM staging system was constructed by replacing the AJCC N category with the LNR category in the AJCC TNM staging system. We evaluated the prognostic relevance of the TNM and TRM staging systems by multivariate analysis.

### Clinicopathological factors

The clinicopathological data collected for subsequent analysis included gender (male or female), age (≤65 years or >65 years), smoking history (none or yes), tumor location (upper, middle, lower), tumor size (≤3.5 cm or >3.5 cm), histological grade (well differentiated, moderately differentiated, poorly differentiated), 7th edition T category (T1: tumor invaded the mucosa or submucosa layer; T2: tumor invaded the muscular layer or the subserosa; T3: tumor invaded the serosa or penetrating serosa; and T4: tumor invaded adjacent organs), 7th edition N category (N0, N1, N2, N3), LNR category (LNR0, LNR1, LNR2, LNR3), TNM stage (I, II, IIIA, IIIB, IIIC) and TRM stage (I, II, IIIA, IIIB, IIIC).

### Follow-up

After curative resection, the patients were followed up according to our standard protocol: every three months for the first two years, every six months during the third to the fifth year, and then annually thereafter until death or the last follow-up date of December 31, 2014. Clinical, laboratory, and imaging examinations were performed in each visit. Endoscopic examinations were performed when necessary. The median follow-up period after surgery for the entire cohort was 30 months (range, 3~108 months). OS was calculated as the time from operation to the date of death or final follow-up.

### Statistical analyses

Statistical analyses were performed with the SPSS 17.0 (SPSS, Inc., Chicago, IL) software and programming language R (version 3.2.2 for Windows). Spearman’s correlation analysis was conducted to investigate the relationships of retrieved LNs with the number of metastatic LNs and LNR. Survival curves and univariate analysis were estimated via Kaplan-Meier method and compared by the log-rank test with GraphPad Prism 5. Three-step multivariate analyses with the Cox proportional hazard model were applied to identify independent prognostic variables^25^. The nomogram was formulated to provide visualized risk prediction on the basis of the results of multivariate analyses by R 3.2.2 with the survival and rms packages. The calibration curves were finally derived through regression analysis.

The predictive discriminatory ability of each staging system was evaluated by using the adjusted HR and AIC. A large HR corresponds to an enhanced system, whereas a small AIC represents a better discriminatory model^26^. To compare the prediction accuracy of each staging system, we calculated the C-index value for each staging system. This index can estimate the probability of concordance between the observed and predicted OS. A large C-index indicates an accurate prognostic prediction (a C-index of 1 represents a prediction accuracy of 100%)^27^. Furthermore, the AUCs of each system for predicting 5-year OS were measured and compared by using the method established by DeLong *et al*.^28^. A two-tailed *P* value of <0.05 was considered statistically significant.

## Additional Information

**How to cite this article**: Zhang, H. *et al*. Metastatic lymph node ratio demonstrates better prognostic stratification than pN staging in patients with esophageal squamous cell carcinoma after esophagectomy. *Sci. Rep.* **6**, 38804; doi: 10.1038/srep38804 (2016).

**Publisher's note:** Springer Nature remains neutral with regard to jurisdictional claims in published maps and institutional affiliations.

## Supplementary Material

Supplementary Information

## Figures and Tables

**Table 1:** Distribution of clinicopathological variables and univariate survival analysis of 387 patients with ESCC by the Kaplan–Meier method.

Variables	No. of patients	5-YSR (%)	HR (95% CI)	Log-rank *x*^*2*^ value	*P* value
Gender			0.910 (0.663–1.249)	0.343	0.558
Male	320	31.5			
Female	67	29.4			
Age (years)			1.295 (1.012–1.658)	4.290	**0.038**
≤65	180	34.2			
>65	207	28.6			
Smoking history			1.441 (1.089–1.908)	4.967	**0.026**
None	116	39.9			
Yes	271	27.5			
Tumor location			0.765 (0.577–1.013)	3.536	0.171
Upper	21	23.8			
Middle	314	30.1			
Lower	52	40.8			
Tumor size			1.859 (1.471–2.499)	24.204	**0.000**
≤3.5 cm	159	43.2			
>3.5 cm	228	23.9			
Histological grade			1.387 (1.081–1.779)	6.625	**0.036**
Well-differentiated	33	49.2			
Moderately-differentiated	294	30.8			
Poorly-differentiated	60	22.5			
T category			1.511 (1.288–1.772)	28.118	**0.000**
T1	42	57.9			
T2	36	36.2			
T3	222	29.4			
T4	87	21.3			
N category			1.641 (1.451–1.856)	68.800	**0.000**
N0	223	42.5			
N1	99	21.9			
N2	35	7.1			
N3	30	3.4			
LNR category			1.718 (1.514–1.950)	85.455	**0.000**
LNR1	223	42.5	43		
LNR2	95	20.1	24		
LNR3	38	12.9	14.6		
LNR4	31	3.6	7.9		

No.: number; 5-YSR: 5-year survival rate; T: tumor invasion; N: node; LNR: lymph node ratio; HR: hazard ratio; CI: confidence interval.

**Table 2:** Multivariate survival analysis of the variables affecting the overall survival of 387 patients with ESCC by a Cox proportional hazard model.

Variables	Multivariate analysis 1			Multivariate analysis 2			Multivariate analysis 3		
	HR	95% CI	*P* value	HR	95% CI	*P* value	HR	95% CI	*P* value
Age	1.258	0.973–1.627	0.080	1.252	0.968–1.620	0.087	1.263	0.975–1.634	0.077
Smoking history	1.299	0.979–1.725	0.070	1.275	0.966–1.684	0.086	1.304	0.984–1.726	0.064
Tumor size	1.703	1.296–2.239	**0.000**	1.749	1.333–2.295	**0.000**	1.781	1.355–2.341	**0.000**
Histological grade	1.321	1.024–1.703	**0.032**	1.323	1.024–1.709	**0.032**	1.328	1.028–1.716	**0.030**
T category	1.359	1.157–1.597	**0.000**	1.394	1.187–1.636	**0.000**	1.410	1.199–1.657	**0.000**
N category	1.513	1.334–1.717	**0.000**	—	—	—	0.845	0.611–1.169	0.309
LNR category	—	—	—	1.662	1.459–1.893	**0.000**	1.944	1.405–2.689	**0.000**

T: tumor invasion; N, node; LNR: lymph node ratio; HR: hazard ratio; CI: confidence interval.

For multivariate analysis 1, all significant factors in the univariate analysis were included, excluding LNR category.

For multivariate analysis 2, N category was replaced by LNR category.

For multivariate analysis 3, both N and LNR categories were included.

**Table 3:** Five-year overall survival of 387 patients with ESCC based on the TNM and TRM stages.

Variables	No. of patients	5-YSR (%)	Univariate analysis		Multivariate analysis	
			*x2*	*P* value (log-rank)	HR (95% CI)	*P* value
TNM stage			78.560	**0.000**	1.522 (1.381~1.678)	**0.000**
Stage I	45	59.5				
Stage II	158	38.7				
Stage IIIA	102	26.0				
Stage IIIB	19	6.0				
Stage IIIC	63	7.0				
TRM stage			100.351	**0.000**	1.580 (1.430~1.746)	**0.000**
Stage I	47	54.5				
Stage II	150	42.2				
Stage IIIA	105	24.8				
Stage IIIB	20	6.7				
Stage IIIC	65	5.3				

No.: number; 5-YSR: 5-year survival rate; TNM: tumor-node-metastasis; TRM: tumor-ratio-metastasis; HR: hazard ratio; CI: confidence interval.

**Table 4:** Comparative survival analysis on the discriminatory ability and prediction accuracy of each staging system for ESCC.

Classification	Figure	Subgroups	HR	AIC	C-index	AUC (95% CI)
N category	2A	N0, N1, N2, N3	1.513	2697.057	0.713	0.717 (0.667–0.768)
LNR category	2B	LNR0, LNR1, LNR2, LNR3	1.662	2690.027	0.721	0.726 (0.676–0.776)
TNM stage	5A	I, II, IIIA, IIIB, IIIC	1.522	2685.089	0.723	0.728 (0.665–0.781)
TRM stage	5B	I, II, IIIA, IIIB, IIIC	1.580	2671.041	0.738	0.744 (0.694–0.794)

N: node; LNR: lymph node ratio; TNM: tumor-node-metastasis; TRM: tumor-ratio-metastasis; HR: hazard ratio; AIC: Akaike information criteria; C-index: concordance index; AUC: area under the receiver operating characteristic curve; CI: confidence interval.
