# Supplementary material for: Metastatic lymph node ratio demonstrates better prognostic stratification than pN staging in patients with esophageal squamous cell carcinoma after esophagectomy
Source: Sci Rep. 2016 Dec 12;6:38804. doi: 10.1038/srep38804 (PMC5150247; doi:10.1038/srep38804)
Supplement: Supplementary Information [file srep38804-s1.pdf]

Metastatic lymph node ratio demonstrates better prognostic stratification than pN staging in patients with esophageal squamous cell carcinoma after esophagectomy

Hongdian Zhang[1, *], Huagang Liang[2, *], Yongyin Gao[3, *], Xiaobin Shang[1], Lei Gong[1], Zhao Ma[1], Ke Sun[1], Peng Tang[1], Zhentao Yu[1]

[1] Department of Esophageal Cancer, Tianjin Medical University Cancer Institute and Hospital, Key Laboratory of Cancer Prevention and Therapy of Tianjin City, Tianjin 300060, China

[2] Department of Thoracic Surgery, The First Hospital of Qinhuangdao, Hebei 066000, China

[3] Department of Cardiopulmonary Function, Tianjin Medical University Cancer Institute and Hospital, Key Laboratory of Cancer Prevention and Therapy of Tianjin City, Tianjin 300060, China

[*] These authors contributed equally to this study

Corresponding author: Zhentao Yu

Supplementary information

Supplementary Table S1. Overall survival rates on the basis of N category according to the LNR category. No.: number; N: node; LNR: lymph node ratio; 5-YSR: 5-year survival rate; MST: median survival time.

Variables	LNR0			LNR1			LNR2			LNR3			*x2*	*P* value
	No.	5-YSR	MST (month)	No.	5-YSR	MST (month)	No.	5-YSR	MST (month)	No.	5-YSR	MST (month)		
N category														
N0	223	42.5%	43	-	-	-	-	-	-	-	-	-	-	-
N1	-	-	-	82	21.9%	26.0	12	34.1%	20.0	5	0	4.9	30.646	0.000
N2	-	-	-	11	10.9%	32.6	17	6.3%	15.1	7	0	9.0	8.028	0.018
N3	-	-	-	2	50.0%	16.0	9	11.1%	13.0	19	0	5.9	8.321	0.016
x2	-			0.568			2.423			1.697				
P value	-			0.753			0.298			0.428				

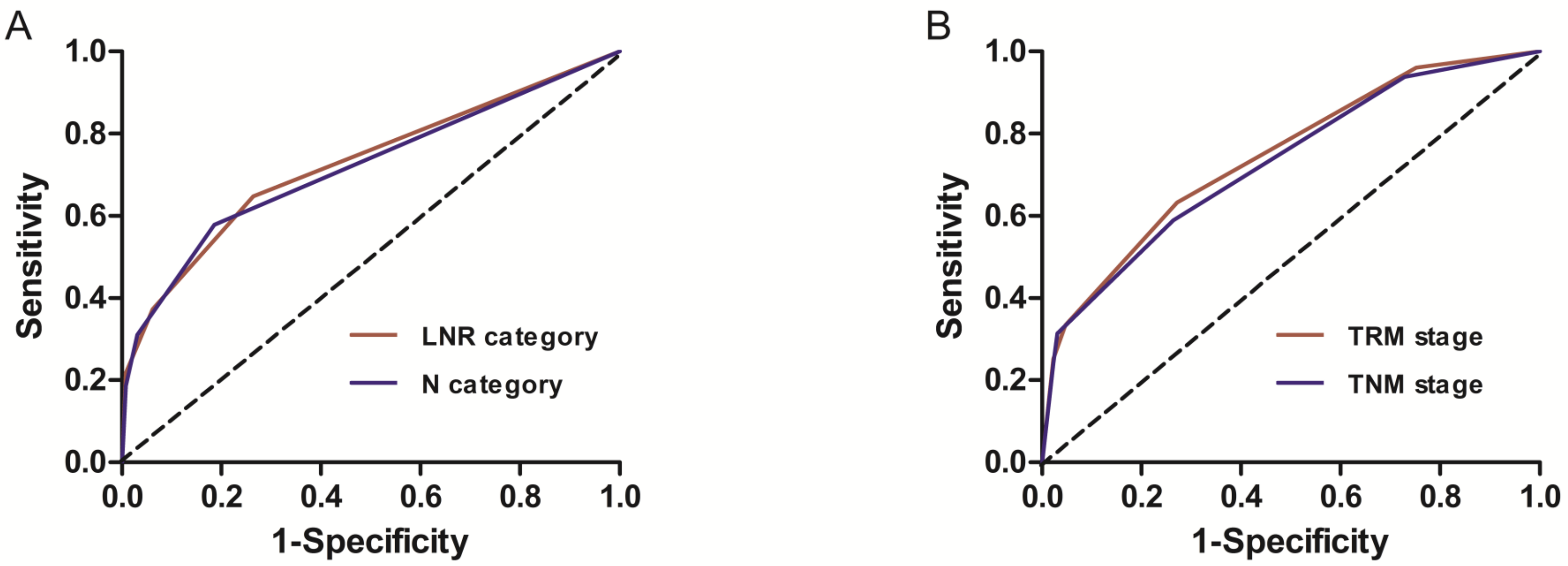

Supplementary Figure S1. Time-dependent ROC curves for 5-year overall survival prediction of ESCC patients after radical esophagectomy. (A) between N category and LNR category, (B) between TNM stage and TRM stage.
